# Advances in the management of gastrointestinal cancers—an upcoming role of immune checkpoint blockade

**DOI:** 10.1186/s13045-015-0185-6

**Published:** 2015-07-16

**Authors:** Gaurav Goel, Weijing Sun

**Affiliations:** Division of Medical Oncology, Department of Medicine, University of Kentucky Markey Cancer Center, Lexington, KY 40536 USA; Division of Hematology-Oncology, Department of Medicine, University of Pittsburgh Cancer Institute, University of Pittsburgh School of Medicine, 5150 Centre Avenue, Pittsburgh, PA 15232 USA

**Keywords:** Checkpoint inhibitors, Gastrointestinal cancers, Immunotherapy, Pembrolizumab, Nivolumab, PD-1, PD-L1, Biomarkers

## Abstract

Gastrointestinal cancers are a group of highly aggressive malignancies, and novel therapeutic strategies with higher clinical efficacy are being actively sought. ‘Immunotherapy’ is now emerging as one such promising strategy for the treatment of these tumors. This article briefly reviews the recent advances that utilize targeting of immune checkpoint pathways, in the management of gastrointestinal malignancies.

## Introduction

Gastrointestinal (GI) cancers are a group of highly aggressive malignancies and a major public health problem worldwide. In the USA alone, 291,000 new cases are estimated to be diagnosed, and approximately 149,000 patients will die from these cancers in 2015 [1]. The currently approved treatments for these tumors result in only modest improvement in overall survival (OS), especially when dealing with advanced disease. Consequently, novel therapeutic strategies with higher efficacy are being actively sought.

Immunotherapy is a relatively new and evolving field of cancer therapeutics that has already demonstrated durable responses in solid tumors including melanoma, non-small cell lung cancer, and renal cancer and is associated with encouraging activity in hematologic malignancies as well [2, 3]. In contrast, the progress made towards development of effective antitumor immune therapies for GI cancers has been relatively slow. GI malignancies usually lack naturally occurring effector T cell responses and have been traditionally considered to be poorly immunogenic. With the identification of new immune-based targets including immune checkpoints, immunotherapy is now beginning to emerge as a promising therapeutic strategy [4, 5]. This article highlights the recent immunotherapeutic advances that were witnessed in the field of GI malignancies.

## Immune checkpoint blockade

It is now well established that tumors evade the host immune response using a multitude of mechanisms, including expansion of immunosuppressive cells (regulatory T [T_reg_] cells, myeloid-derived suppressor cells [MDSCs]) in the tumor microenvironment, elaboration of various cytokines and chemokines (transforming growth factor-β [TGF-β], interleukin [IL]-10, indoleamine 2,3-dioxygenase [IDO]), and co-inhibitory signaling pathways mediated via immune checkpoints (cytotoxic T lymphocyte-associated protein-4 [CTLA-4], programmed cell death-1 [PD-1], T cell immunoglobulin- and mucin-domain-containing molecule-3 [TIM-3], and lymphocyte activation gene 3 [LAG3]) [6]. Together, these contribute to the development of resistance to immune effectors.

Immune checkpoint blockade strategy is now being actively evaluated in the management of GI malignancies including esophageal, gastric, colorectal, and liver cancers. PD-1, which is a co-inhibitory receptor expressed on the surface of activated T cells, B cells, and myeloid cells, interacts with its ligands (PD-L1 and PD-L2) to prevent T cell functioning. Antibody mediated blockade of PD-1 or PD-L1 results in inhibition of this checkpoint, leading to T cell functional activation and enhanced antitumor activity. Emerging data suggest encouraging activity of PD-1 axis blockade in the management of GI cancers.

## Gastric and esophageal carcinoma

The Cancer Genome Atlas (TCGA) project performed comprehensive molecular characterization of gastric adenocarcinoma and identified four major molecular subtypes, namely, Epstein-Barr virus (EBV)-infected tumors, microsatellite instability (MSI) tumors, genomically stable tumors, and chromosomally unstable tumors [7]. In the EBV subgroup, there was amplification at 9p24.1 leading to upregulation of PD-L1 and PD-L2, which indicates a potential role of PD-1 axis blockade in treatment of these patients.

KEYNOTE-012 is a multi-center, multi-cohort, non-randomized phase Ib trial evaluating the safety and efficacy of anti-PD-1 antibody pembrolizumab (MK-3475) in patients with previously treated, PD-L1-positive, advanced cancers (NCT01848834) [8]. Patients were classified as PD-L1 positive based on ≥1 % of tumor cells demonstrating expression of PD-L1 marker, or any positive staining in the tumor stroma. In the gastric cancer cohort, 39 patients who were previously treated for their metastatic diseases have been enrolled. The observed median duration of response (DoR) was 24 weeks. The 6-month progression free survival (PFS) and OS rates were 24 and 69 %, respectively. PD-L1 expression level was found to correlate with the objective response rate (ORR; *P* = 0.10). In this study cohort, four patients experienced grades 3–5 drug-related adverse events (DRAEs), which included peripheral sensory neuropathy, fatigue, decreased appetite, hypoxia, and pneumonitis. In another similar multi-cohort, phase Ib trial (KEYNOTE-028) of pembrolizumab monotherapy for PD-L1-positive advanced solid tumors, 23 patients with either squamous cell carcinoma (SCC; 74 %) or adenocarcinoma (22 %) of the esophagus or gastroesophageal junction (GEJ) have been treated at the time of interim analysis (NCT02054806) [9]. The ORR was 30 % (40 % for adenocarcinoma, 29 % for SCC). The median DoR was 40 weeks, with 6 of 7 responses ongoing at cutoff. DRAEs were observed in 39 %, including grade 3 toxicity (lymphopenia, decreased appetite, liver disorder, pruritic rash) in 17 % (*n* = 4) of the patients.

In summary, the available results from KEYNOTE-012 and KEYNOTE-028 demonstrate a promising clinical activity of pembrolizumab monotherapy in heavily pretreated, PD-L1-positive, advanced gastric and esophageal carcinoma, respectively.

## Colorectal cancer

MSI tumors are characterized by epigenetic silencing or mutations of DNA mismatch repair (MMR) genes, leading to the formation of variable length DNA microsatellites. The high mutational load in MSI tumors creates many tumor-specific neoantigens, some of which are recognized as foreign by T cells, and this contributes to the lymphocytic reaction frequently observed in these tumors [10]. MSI colorectal cancers (CRCs) comprise approximately 15 % of the total sporadic CRCs, and these tumors have been shown to upregulate expression of immune checkpoints including PD-1, PD-L1, CTLA-4, LAG-3, and IDO in tumor infiltrating lymphocytes (TILs), stroma or tumor invasive front compartments [11].

A phase II study evaluated the clinical activity of pembrolizumab monotherapy in patients with previously treated, progressive metastatic tumors, with and without MMR deficiency (NCT01876511) [12]. The patients were enrolled in three cohorts, MMR-deficient CRC (*n* = 11), MMR-proficient CRC (*n* = 21), and MMR-deficient non-colorectal tumor cohort (*n* = 9). The study met its co-primary endpoints of immune-related ORR (irORR) and immune-related PFS (irPFS) at 20 weeks, for both the MMR-deficient cohorts. The irORR and irPFS were 40 and 78 % for MMR-deficient CRC, 0 and 11 % in the MMR-proficient CRC cohort. Response rates (RR) and disease control rates (DCR) were 40 and 90 % in MMR-deficient CRC, 0 and 11 % in MMR-proficient CRC cohort. Median PFS and OS were not reached in the MMR-deficient CRC group but was 2.2 and 5.0 months in the MMR-proficient CRC cohort (hazard ratio [HR] for PFS = 0.103; 95 % confidence interval [CI], 0.029 to 0.373; *P* = 0.001 and HR for OS = 0.216; 95 % CI, 0.047 to 1.000; *P* = 0.05). These results provide promising evidence to suggest that MMR status predicts clinical benefit from immune checkpoint blockade in advanced CRC.

## Hepatocellular carcinoma

The liver is characterized by the presence of an immunosuppressive microenvironment and expression of PD-L1 on sinusoidal endothelial and Kupffer cells. Anti-PD-1 blockade strategy has demonstrated manageable toxicity profile and preliminary evidence of efficacy in a recently reported study involving advanced hepatocellular carcinoma (HCC) patients [13]. This multiple ascending-dose phase I/II study evaluated the safety and antitumor efficacy of nivolumab in patients with pretreated or sorafenib-intolerant advanced HCC (NCT01658878). Patients with Child-Pugh score ≤B7 were enrolled in three independent parallel cohorts, namely, uninfected, HCV-infected, and HBV-infected cohorts. DRAEs occurred in 32 patients (68 %; 19 % grades 3–4) and included elevation of lipase, amylase, aspartate aminotransferase (AST) and alanine aminotransferase (ALT), rash, anemia, and fatigue. No maximum tolerated dose (MTD) was identified in any cohort. At the time of the interim analysis in March 2015, response was evaluable in 42 patients and included two complete responders (CR; 5 %) and six partial responders (PR; 14 %). Objective clinical responses were observed in both uninfected and viral-associated HCC. The OS rate was 70 % at 9 months and 62 % at 12 months. Responses were ongoing in six of eight patients at the time of interim analysis. Patient enrollment is continuing in the dose expansion phase of the study.

## Pancreatic cancer

Checkpoint pathways are also being evaluated as immunotherapeutic targets for the treatment of pancreatic cancer. However, due to an immunologically quiescent microenvironment associated with these tumors, none of the examined approaches have demonstrated a clinically meaningful benefit till date [14]. CD40 activation can reverse immune suppression and drive antitumor T cell responses. Agonist CD40 antibody has been used in combination with gemcitabine in a phase I study to shrink pancreatic ductal adenocarcinoma by stimulating tumor macrophages against pancreatic cancer stroma [15]. Single agent ipilimumab was evaluated in a phase II trial of advanced pancreatic cancer and failed to demonstrate an appreciable antitumor activity [16]. The combination of ipilimumab with gemcitabine in advanced pancreatic cancer is currently under phase Ib evaluation (NCT01473940). A phase Ib/II trial is studying the safety and immunological effect of neoadjuvant chemoradiation therapy (CRT) added to pembrolizumab in resectable or borderline resectable pancreatic cancer (NCT02305186). Other strategies that are being evaluated include combination of vaccine (GVAX, CRS-207) with antibody against CTLA-4 (NCT01896869) or PD-1 (NCT02243371).

## Future directions, challenges, and conclusion

The potential of immunotherapy as a treatment modality for GI malignancies has finally become a reality. However, the clinical benefit from these agents is restricted to only a subset of patients, at least at the present time. Moreover, these immune-modulating therapies are often associated with immune-mediated toxicities. Consequently, biomarkers are needed to refine patient selection for optimum clinical benefit and minimize toxicity [17].

PD-L1 positivity has been used to select patient population in KEYNOTE trials. Experience from a series of clinical trials across a variety of tumor types suggests that although PD-L1 positivity is associated with a greater likelihood of response from anti-PD-1 or anti-PD-L1 agents, the benefit is not restricted to the PD-L1-positive population exclusively [18]. Currently, the evaluation and validation of PD-L1 positivity as a predictive biomarker suffers from several limitations including lack of standardized definition for PD-L1 positivity, lack of uniformity in antibody clone used for immunohistochemistry staining across studies, and discordance between primary tumor and metastatic lesions [18].

The presence of MSI is a potential genetic biomarker shown to have a predictive value in the study presented by Le and colleagues [12]. Considering the fact that majority of GI tumors are microsatellite stable (MSS) and therefore immunogenically quiescent, additional approaches that utilize combination of immune checkpoint inhibitors with T cell-inducing strategies such as use of vaccines, chemotherapy, targeted agents, or radiotherapy need to be evaluated to continually improve upon the existing responses.

To conclude, immune therapy using checkpoint blockade has added a new treatment paradigm in the management of GI malignancies. Further evaluation with future clinical trials that enroll larger patient cohorts and study combinatorial approaches is strongly warranted.
